# An open-source natural language processing toolkit to support software development: addressing automatic bug detection, code summarisation and code search

**DOI:** 10.12688/openreseurope.14507.1

**Published:** 2022-03-14

**Authors:** Cristian Robledo, Francesca Sallicati, Gaël de Chalendar, Marcos Fernández, Pablo de Castro, Eduardo Martín, Javier Gutiérrez, Yannis Bouachera

**Affiliations:** 1 Tree Technology, Llanera, Asturias, Spain; 2 CEA, Paris, Île-de-France, France

**Keywords:** Natural Language Processing, Variable Misuse, Code Summarisation, Semantic Parsing, Deep Learning, Software Engineering

## Abstract

This paper aims to introduce the innovative work carried out in the Horizon 2020 DECODER project – acronym for “DEveloper COmpanion for Documented and annotatEd code Reference” – (Grant Agreement no. 824231) by linking the fields of natural language processing (NLP) and software engineering.

The project as a whole addresses the development of a framework, namely the Persistent Knowledge Monitor (PKM), that acts as a central infrastructure to store, access, and trace all the data, information and knowledge related to a given software or ecosystem. This meta-model defines the knowledge base that can be queried and analysed by all the tools integrated and developed in DECODER. Besides, the DECODER project offers a friendly user interface where each of the predefined three roles (i.e., developers, maintainers and reviewers) can access and query the PKM with their personal accounts.

The paper focuses on the NLP tools developed and integrated in the PKM, namely the deep learning models developed to perform variable misuse, code summarisation and semantic parsing. These were developed under a common work package – “Activities for the developer” – intended to precisely target developers, who can perform tasks such as detection of bugs, automatic generation of documentation for source code and generation of code snippets from natural languages instructions, among the multiple functionalities that DECODER offers. These tools assist and help the developers in the daily work, by increasing their productivity and avoiding loss of time in tedious tasks such as manual bug detection.

Training and validation were conducted for four use cases in Java, C and C++ programming languages in order to evaluate the performance, suitability, usability, etc. of the developed tools.

## Plain language summary

Software engineers usually spends a lot of time in tedious activities like debugging and documenting code or finding examples of code snippets to use as a basis for their new programmes. Given the large and complex software systems that exist nowadays, being forced to perform these tasks manually causes a considerable drop in the overall productivity of programmers. The models developed in this work target Java, C and C++ programming languages and aim to alleviate software developers’, maintainers’ and reviewers’ efforts, by proposing automatic NLP solutions to carry out tasks such as bug detection, documentation generation and code search.

## 1. Introduction

We live in a world where many important aspects of our daily lives rely on software. As a consequence, it is crucial for software to be of good quality to be maintainable or updated. However, it has been estimated that developers lose about 60% of their productivity ^1^ in struggling to understand badly written code or in tasks like detecting bugs or searching the Internet for documentation or snippets of code.

Recently, research groups started to advance in the application of machine learning in the field of software engineering, by adapting deep learning architectures to suit the needs of software engineering tasks, finding solutions to assist software engineers in tasks like automatic repair, code completion, code search and code summarisation among others.

This paper focuses on the work carried out in the DECODER project, which aims to improve the productivity of IT professionals. This project developed a set of NLP-based tools to automatically generate code, detect bugs and perform code summarisation and code search, with the objective of supporting developers in their day-to-day work for the selected languages (Java, C and C++).

Finding bugs in source code is a core problem in software engineering and programming language research. The challenge in this domain lies not only in correctly characterising source code that contains a bug with high precision, but also being able to correct it. Among all the possible errors that can be found, those that affect the correct behaviour of the software are of especial interest. An example of this kind of bug is variable misuse (VarMisuse) ^2^, which refers to the wrong use of variables and is perhaps one of the most common errors that cause programme breaks: given a programme, a VarMisuse bug exists when a correct variable differs from the current one at a certain location. These types of errors may occur when, for example, a programmer copies some code into a new context but forgets to rename a variable from the old context, or when two variable names within the same scope are easily confused.

Many people may think that repairing variable misuses is a trivial task that can be done manually. However, identifying the locations of faults in source code has been recognised to be tedious ^3^, given not only the necessity of a rich background knowledge and complex logical reasoning about the original programmer’s intent, but also the size of large-scale software systems today. Thus, in order to alleviate the workload concentrated on debugging variable misuse bugs, we present an approach that takes advantage of deep learning sequence-to-sequence models to locate and repair a wrong use of variables in source code files, as well as classify programmes as correct or faulty.

Another crucial activity within the software development lifecycle is documenting source code. Often times, large software repositories lack a proper documentation, which can be imprecise, vague, outdated or even missing. It is well-know that good code summaries can help in avoiding time loss and that they are essential to improve code comprehension and code search, especially during maintenance or evolution of a software project ^4^. Thus, generating natural language description for source code can positively impact on developers’ daily work, facilitating a tool that works in synchrony with them in the monotonous task of documenting snippets of code.

In the last few years, a consistent number of solutions to achieve the task of code summarisation were already proposed, especially working with Java. The common basis for these approaches typically consists of using encoder-decoder neural networks, being usually sequence-to-sequence or transformer models. Based on 5, our approach introduces widely used programming languages such as C and C++ in the context of code summarisation, achieving good performances with the usage of transformers models. 

As we mentioned previously, these tools aim to make easier the workflow of IT professionals, independent of whatever their role in a software development project. They lie on the idea of getting developers familiar not only with the code but also with a whole project in a simple and fast way. However, recent works go beyond this concept nowadays, such as semantic parsing.

Semantic parsing consists of transforming natural language into more formal ones like programming languages. Therefore, it can be seen as the reverse task of code summarisation. Recent works inspired by sequence-to-sequence processing propose architectures based on encoder-decoder neural networks whose decoder is constrained to conform to the grammar of the target language. Such techniques have already been experimented to generate code from a description in natural language ^6^. In this work, we improve existing models with the latest developments in NLP and also apply them on a new language pair, namely the pair *{natural language, C++}*.

The paper is structured across five main sections: Section II presents the most recent works and state-of-the-art models related to the three NLP tools, emphasising those techniques that are based on deep learning architectures. Section III details all the methods adopted for building each model in terms of data, pre-processing techniques and architectures selected for the final deployment in the DECODER framework. Section IV covers the results obtained, providing commonly used metrics to assess performances for each tool, as well as displaying some concrete application examples. Finally, Section V sums up the achievements reached with these tools and gathers possible approaches for future research in this field.

## 2. Related work

### 2.1 Variable misuse

Within the automated program repair (APR) field, several works have focused on the variable misuse problem. Most of them present learning-based repair solutions that learn how to fix this type of error directly from source code examples.

The work already mentioned in 2 introduces the VarMisuse problem. This is addressed by using a graph neural network on syntactic and semantic information to make individual predictions for each variable use in a program and reporting back all variable discrepancies above a threshold.

In 7, a neural model for semantic code repair – where one of the classes of bugs is variable replace (VarReplace) – is presented. This is similar to the VarMisuse problem. In particular, VarReplace refers to an incorrect local variable that is used at a particular location and should be replaced with another variable from the snippet. This framework adopts a two-stage approach where first a large set of repair candidates are generated by rule-based processors, and then these candidates are scored by a statistical model using a novel neural network architecture to select the best one.

Finally, 8 presents an approach that, unlike previous studies, jointly and directly localises and repairs variable misuse bugs, and classifies the programme as faulty or correct. To achieve this, the authors use a novel multi-headed pointer network where two pointers are trained: the first pointer corresponds to the location of the bug, and the second pointer corresponds to the location of the repair variable.

Our approach is also based on this last work and makes use of pointer networks to detect and fix wrong uses of variables. In essence, it could be seen as an extension of the original method, which just targets programming languages like Python (RRID:SCR_008394) and C#. We have been able to extend the approach to other programming languages historically more used – according to the TIOBE programming Community Index – as Java, C and C++ with good results.

### 2.2 Code summarisation

Although in the last few years, research in code summarisation tasks offered many innovative solutions, often based on deep learning models, this kind of applications still remains quite unexplored and usually involves few programming languages. For instance, a huge proportion of papers published on the topic use Java (few use Python) as the object of study.

One example is the model proposed by 5, consisting of a transformer-based architecture that is fed with source code token-level information, rather than working with complex representations like path sequences from the programmes’ abstract syntax trees (ASTs). The results reported in this study represent a significant improvement over previous studies, especially for the summarisation of methods written using Python.

Other experiments in Java come from 9 and 10. Both studies are focused on code summarisation tasks but propose a different approach. In the first paper, they propose DeepCom, a sequence language model, where information is fed from ASTs, with the introduction of a new structured-based traversal method to help keep sequences unambiguous and reversible to its original form. In the second study, TLCodeSum is presented, consisting in an attention sequence-to-sequence model that incorporates previously learned information from API sequences as an additional encoder. The aim is to produce natural language description for snippets of code.

In 11, ASTs are also employed as the input layer for a new proposed type of Tree-LSTM (long short-term memory) model attention model. Since standard Tree-LSTMs cannot handle a node that has an arbitrary number of children and their order in ASTs simultaneously, the authors developed an extension of Tree-LSTM, which they called Multi-way Tree-LSTM, to be set as the encoding layer for their network, handling such representations.

Another approach involving Python is proposed by 12. This model uses both AST extracted sequences and token-level information to feed a deep reinforcement learning model, which instead of using a simple decoder to greedily predict the next most probable correct word, introduces at each time step an actor and a critic network that jointly select the best candidate word.

By further investigating the state of the art, few studies involving other programming languages can be found, apart from a couple of papers that focus of SQL and C#. Developed under the framework of the DECODER project, our approach on code summarisation involves a transformer-based architecture inspired by 5, where the main novelty consists in the implementation of these techniques not only for the Java language but also for C and C++ programming languages, which are widely used languages, even though they have not been used in the context of automatic documentation generation for source code.

### 2.3 Semantic parsing

Translating natural languages into formal languages is called Semantic Parsing. It has been applied to the generation of formal languages like λ-calculus ^13^ or the abstract meaning representation (AMR) ^14^. It has also been largely used to help user query databases by converting their requests into SQL ^15^ or other kinds of instructions ^16, 17^. Moreover, semantic parsing is also used to generate programming languages like Python ^18^ and Java ^19^. Recently, BERT-based models have been designed, like RecycleBERT ^20^, a transformer model whose encoder has been replaced by pre-trained BERT ( **RRID:SCR_018008)**.

In semantic parsing, target languages are formal ones respecting a strict grammar. Several works have tried to take into account this fact. For example, 15 filters out invalid generated SQL queries. 21 generates a sequence of grammar derivation steps and grammatical constraints. Rabinovich ^18^ uses a more abstract representation, the abstract syntax network (ASN), which encodes ASTs represented with the generic abstract syntax description language (ASDL) framework ^22^. The already mentioned TRANX ^6^ uses this approach to generate programming languages, SQL and Python.

Our goal in this work is to use jointly RecycleBERT and TRANX to generate accurate and valid Java and C++ code in the framework of the DECODER environment.

## 3. Methods

The tools presented in this work are available from GitHub and archived with Zenodo ^23– 25^.

### 3.1 Data

***3.1.1. Use cases datasets***

DECODER datasets include four uses cases, whose main features are presented in Table 1 and can be found as *Underlying data* ^26– 28^. Note that these datasets represent a common basis for the development of the models involved in this work. For the variable misuse model, bugs were automatically generated for the source code methods according to the rules described in the following section, whereas the natural language descriptions for the code summarisation model were already provided by the consortium partners together with the code.

**Table 1.** DECODER use cases datasets overview.

Use Case	Leader	Programming Language	Files
Drivers	SYSGO	C	317
OpenCV	TREE	C++	593
MyThaiStar	CAPGEMINI	Java	471
Java	OW2	Java	7,553

*Drivers* and *OpenCV* use cases collect source code files written in C and C++ respectively. The former use case, led by SYSGO, consists in a collection of Linux drivers source code, while TREE *OpenCV* use case gathers programmes belonging to a human-robot interaction application.

Java source code files come from *MyThaiStar* and *Java* use cases. The first use case, led by CAPGEMINI, is an application that manages orders and reservations for an Asian restaurant, while the OW2 *Java* use case brings together four independent projects (Joram, Lutece, Sat4J and Authzforce projects).

It is worth noting that C and C++ files were merged to develop both Variable Misuse and Code Summarisation models, due to the scarcity of available training data. Therefore, from here on we will refer to C/C++ and Java models/use cases.

***3.1.2. Datasets for variable misuse***

Focusing on the VarMisuse task, the need to augment the training set with publicly available code from GitHub was needed for the C/C++ model, due to the scarcity and quality of the data covering these two programming languages. As Table 1 displays, there are only 911 (319+592) source code files provided by the Drivers and OpenCV use cases, respectively, which would be an important limitation. After the search on GitHub, the number of source code files increased in 1,618 files.

***3.1.3 Datasets for code summarisation***

Concerning the code summarisation implementation of the augmented model versions, two datasets are involved:

- Java *DeepComm* ^9^ dataset that gathers about 600k pairs of Java methods and associated NL description, which were already selected and processed by the authors of the paper.
- The augmented C/C++ dataset, built by extracting 250k observations in C and 250k observations in C++ from a SQL database provided by 29. They collected source code files from GitHub repositories written in C, C++, Java and Python; extracted comments using Doxygen and condensed such pairs into the database. Since the data were not previously filtered or crafted for direct training, the augmented dataset has been selected with a minimum length for the natural language description to avoid considering empty descriptions.

***3.1.4. Datasets for semantic parsing***

For the generation of JAVA, we based our experiments on the Concode corpus ^30^. For C++, we used the DECODER OpenCV use case corpus presented above and a larger but less clean corpus, from the “Code and comments dataset” ^29^, of substantial size, named “C&C” below. This dataset contains a total of 16,115,540 pairs of comment and code, mined from 106,304 GitHub projects coded in Python, Java, C and C++ ^31^.

### 3.2 Data preparation, preprocessing and feature extraction

***3.2.1 Variable misuse***

**Dataset preparation**

An important aspect of the models to be developed for training and evaluating this variable misuse tool is that they have to be presented with both buggy and non-buggy files so as to be able to tell them apart. Assuming that all the code provided has no mistakes regarding the use of the variables, we should generate synthetic datasets in which the bugs are created programmatically as follows:

- The first step is to tokenise all the different source code files and identify all the variables for every snippet.
- After this initial phase to identify all the variables, the next step is to flag those variables that appear more than once for a given snippet as candidates for introducing a bug. This presents the second assumption made by the tool, by which we are assuming that the variables for fixing a bug in a certain snippet should be contained within the same snippet – meaning that the snippet contains at least two different variables. Therefore, we are only introducing bugs in the position of a variable repeated within the same source code file. The candidates for replacing the original variable are all the rest of the variables existing in the same file, and one of them is picked at random for every snippet.
- We iterate over all variable slots within a snippet in order to obtain as many buggy files as repeated variables locations exist. In addition, we include a copy of the original file for each buggy file created with the objective of ensuring a 50/50 balance between buggy and non-buggy files.
- Finally, it is important to highlight that we are only considering that each of the synthetic files created only contains a single bug per file.

Since the new application proposed can be stated as a classification problem, the models to be implemented in this tool need to receive the corresponding labels. Here, two different labels are used and included to the tokenised files as vectors, one of them is called “location”, whose purpose is to mark the position of a token in a source code considered as buggy. This vector, which has the same length than the programme, is 1 at the location containing bug and 0 otherwise. In case the programme does not contain any variable misuse bugs, the location vector will point to the first position or token of it. The other label is called “repair” and marks all the occurrences of the variable that fixes a bug within the same file. As a result, this second vector contains all 0 except for the positions of the correct variable for the location of the bug, which should be 1. Again, if there is no error in the file, the repair vector will not point to any position of the programme.

By following this process, we have created two different datasets: one for those programmes written in Java, and one for C and C++ files. For this latter case, a first tokenisation of the available source code files showed that most of them were too long, so it was decided to extract just the methods contained on each file with the aim of continuing to increase the number of data available for the model. Table 2 shows the number of available source code files for each model, as well as the number of created buggy files associated with them. It is important to note that the number of files used by the model doubles the number of these buggy files due to the goal of a 50/50 balance with non-buggy files.

**Table 2.** Generated datasets for variable misuse.

Use Cases	Number of files	
	Original files	Created buggy files
Java	8,058	54,448
C/C++	2,539	29,166

**Preprocessing and feature extraction**

Besides, before feeding them to the models built, programmes need to be pre-processed so that models can consume them. In this case, the source code files are processed according to the following steps:

- First of all, programmes must be tokenised in order to represent them as a token sequence. This tokenisation step is done using Pygments, a tool which offers a variety of lexers for the most popular programming languages to split the source into tokens. These tokens are accompanied by their corresponding type, that determines what the text represents semantically (e.g., keyword, string, or comment).
- After having parsed the source code files, tokenised programmes are filtered by the number of tokens they contain. In this case, we filtered files that contain less than 200 tokens.
- In the next step tokens are mapped to a numerical representation, allowing for constructing a vocabulary for each use case before training phase.
- While building the vocabulary, a <UNK> (‘Unknown’) token is added for those words not present in the training set, which, otherwise, would not have a representation in the validation/test sets or in a new predicting setting.
- As a last step, those sequences that are shorter than the number of tokens established in the previous filtering must be padded. Additionally, sequences that exceed that value must be truncated at the end.

***3.2.2. Code summarisation***

As already mentioned, the data coming from either DECODER use case leaders or augmented open-source datasets already came with natural language description associated with source code methods. Therefore, no further data preparation is needed as a preliminary step to perform source code summarisation.

**Preprocessing and feature extraction**

The essential steps to follow to preprocess the data resembles the previous points used for VarMisuse, with some modifications that are applied to adapt to this task. The main points in this setting are listed below:

- Tokenisation into units of interest and creation of the training vocabularies. In this work the methods’ tokenisation was done with Pygments, before further tokenisation of variables is applied to split identifiers according to naming conventions adopted by developers, such as snake_case and CamelCase. On the other hand, the natural language descriptions were tokenized with a traditional tokeniser.
- Addition of <START> and <END> tokens to the target sequences to facilitate the decoder block to process them.
- Conversion to numerical labels using dictionaries, where an <UNK> label is assigned to words that are not represented in the training set. This is particularly important in inference settings, since words that were not previously known by the models can be assigned to this predefined label.
- Extraction of sequences of equal maximum length, by either padding shorter sequences with 0s or truncating longer sequences to such maximum value. The maxim length has been fixed for code summarisation models at a length of 200 tokens for the input source code and to 15 tokens for the natural language descriptions.

Therefore, the type of information flowing into the networks are token-level sequences derived from both the source code methods and the corresponding natural language descriptions of their functionality, extracted with the above-mentioned tokenisers. This implies that two vocabularies are also involved, representing respectively the set of training tokens for methods and descriptions, which result from the preprocessing techniques and feature extraction methods mentioned in the list above.

***3.2.3. Semantic parsing***

The Java corpus, the Concode one, was already prepared and did not necessitate other changes. Particularly, the corpus is already split into train, development and test sets.

For C++, the related data were retrieved from the C&C corpus. After preprocessing to eliminate duplicates and missing values, the final size of the corpus is then 150,000 comment and code pairs. However, the natural language part of the corpus consists of comments and not of real instructions or exact descriptions, which makes the corpus less suitable for a task such as code generation, as not all information about the developed function is given. Moreover, the corpus is very noisy at the comments level: some of them do not bring any information about the code and simply indicate a potential bug or the necessity to modify the code.

### 3.3. Model implementation

***3.3.1 Variable misuse***

Our architecture is based on the work in 8. As briefly explained in Section 2, this approach allows performing joint prediction of both the location and the repair for VarMisuse bugs. In essence, this model is similar to an encoder-decoder model that combines a long short-term memory (LSTM) ^32^ recurrent neural network with pointer networks.

Given a programme token sequence, first the proposed model embeds the tokens using a trainable embedding matrix. Then, as an encode step, it runs a LSTM over the token sequence to obtain hidden states for each embedded programme token. It is at this point where our model differs from the original one, as it does not use a masking vector to only consider those hidden states that correspond to states of the variable tokens. Therefore, these encoder states are directly used to train two pointers corresponding to the location of the bug and the location of the repair variable. This pointer mechanism is proposed in 33, and is a very simple modification of the attention model that allows applying the method to problems where the output dictionary size depends on the number of elements in the input sequence and whose outputs are discrete and correspond to positions in the input. Since the output of this approach is a softmax distribution, these pointers can essentially be described as distributions over the programme tokens.

**Figure 1.**
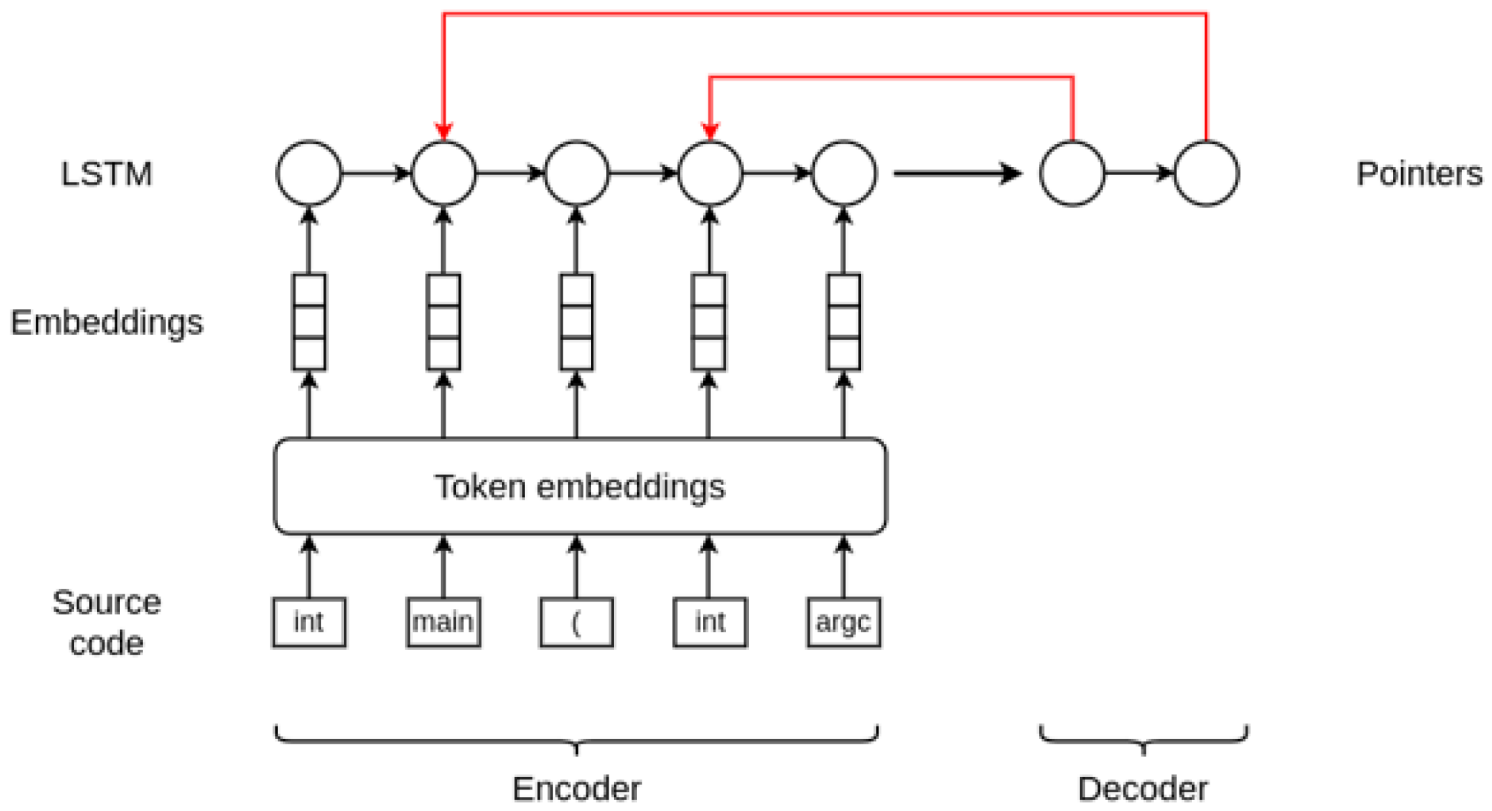
Variable misuse bug detection and repair architecture.

Figure 1 illustrates and summarises the architecture of the model and how it works.

***3.3.2 Code summarisation***

**Figure 2.**
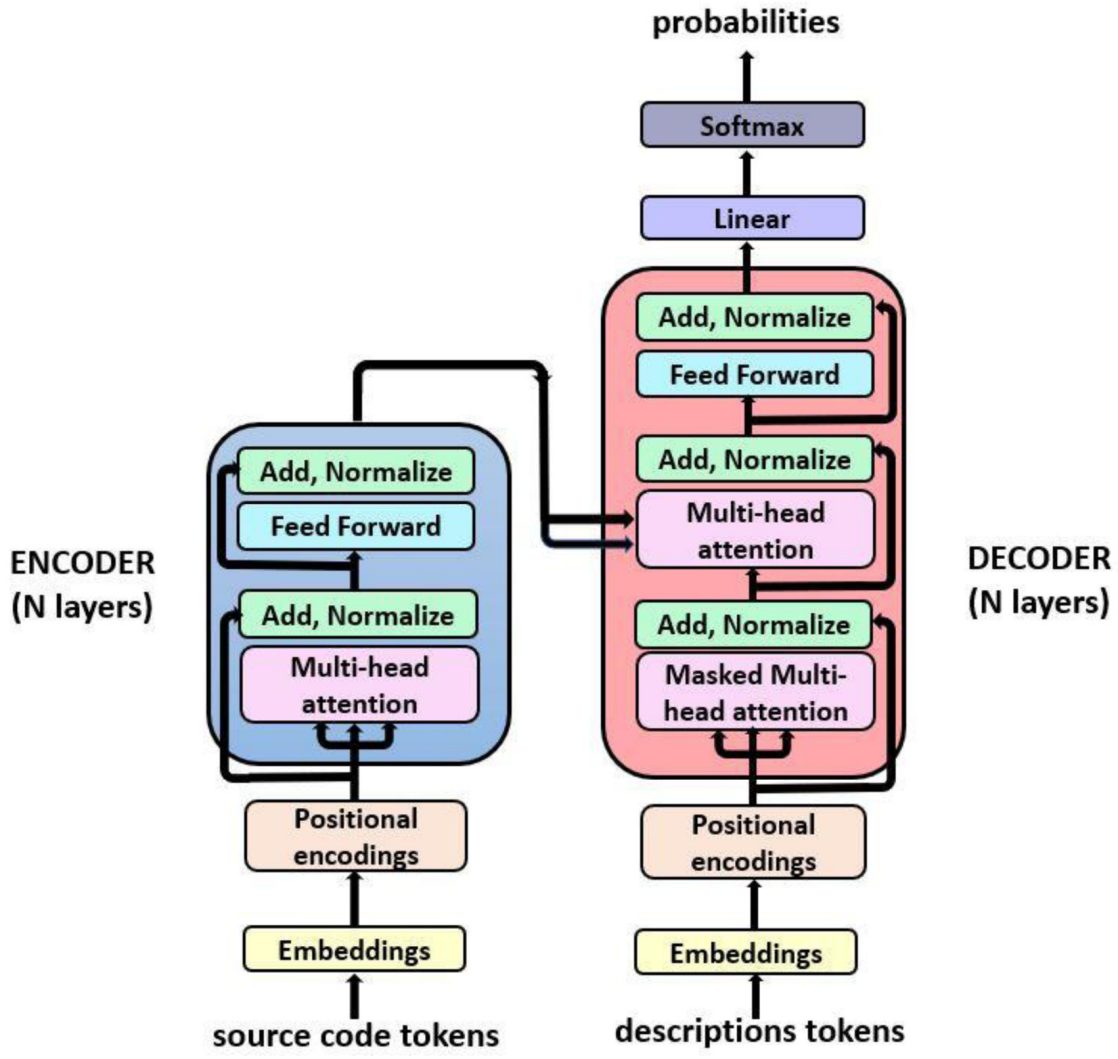
Transformer architecture.

The transformer language model architecture illustrated in Figure 2 was introduced in 2017 in the paper ‘Attention is all You Need’ ^34^ and rapidly became a state-of-the-art model to solve a variety of NLP tasks, such as neural machine translation and text generation among others. This architecture substituted traditional recurrent neural networks (RNNs) architectures, such as LSTMs and gated recurrent units (GRUs) ^35^, which were widely used in NLP tasks, due to numerous advantages such as its ability to learn long-range dependencies without assuming temporal/spatial relationship across input data. Moreover, transformers proportion a huge benefit in terms of scalability, due to parallel computing capabilities.

Our model consists in an implementation of a transformer model, based on ^5^. The model is composed of two main parts: the encoder and the decoder, which are blocks of encoder/decoder layers stacked on the top of each other. The internal configuration for the encoder and decoder is a combination of multi-head attention and feedforward layers. The main features involved in this architecture are:

- **Positional encoding**: Information added to the embedding vector regarding the tokens position in the sentence. This way, words will be closer to each other in the space based on both words meaning similarity, thanks to the embedding vectors, and their position in the sentence due to the positional encoding of tokens.
- **Masking**: This mechanism consists in a binary vector which is used as an indicator of which tokens should or should not be processed.
- **Multi-head attention**: Each multi-head attention block receives three inputs; Query, Key and Value, which are put through dense layers and split up into multiple heads. This allows the model to jointly pay attention to the information at different positions from different representational spaces.

***3.3.3 Semantic parsing***

Several models were trained and evaluated on the Concode and C++ corpora:

- A classical transformer model, coded using the *nn.Transformer* module of PyTorch ( **RRID:SCR_018536)**, and trained with the classical PyTorch training procedure;
- CONCODE’s encoder-decoder model. However, we were not able to reproduce its results. The model has not been updated for several years and the instructions on the project’s repository were unclear about the versions of the modules required to work correctly;
- TRANX, in order to evaluate the performance of a model generating syntactically correct code. An adaptation of the TRANX code to JAVA generation was necessary at the level of the ASDL grammar and the parser;
- RecycleBERT to evaluate the performance of a translation model using BERT embeddings, and thus compare its performance with that of a classical transformer;
- A modified version of RecycleBERT in which the encoder is not a pretrained BERT model but a pretrained CharacterBERT ^36^ model, still for comparison purposes. CharacterBERT replaces the BERT wordpiece tokenisation by character-based embeddings based on ELMO model. It should allow to better handle out of BERT vocabulary tokens which are numerous in comments and even more in code.

**Figure 3.**
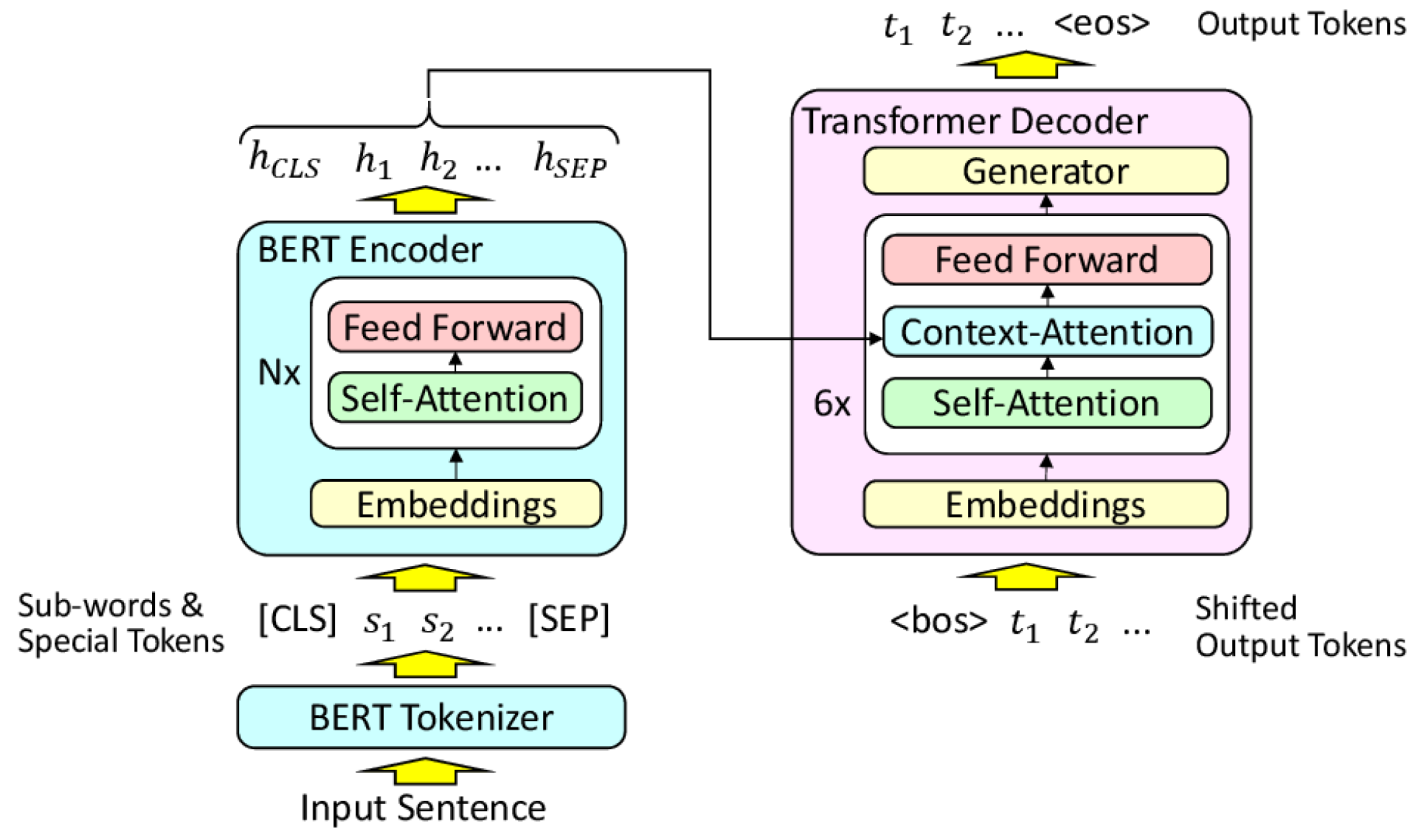
RecycleBERT architecture (From [recyclebert], under CC-BY). [CLS] and [SEP] are two special tokens added before and after the source tokens si. hj are tensors for each token at the output of the encoder. <bos> is a special token added at the beginning of the tk target output tokens and . Likewise, <eos> is a special token generated at the end of the sequence of generated output tokens.

RecycleBERT is a transformer model whose encoder has been replaced by pretrained BERT. Unlike the usual monolingual tasks in which BERT excels (named entity extraction, classification...), translation still requires an encoder-decoder structure. Finetuning BERT on a translation task would be like applying this finetuning operation to a transformer model whose encoder is in fact a pretrained BERT. However, unlike the classical case where a single layer with relatively few parameters is placed at the output of BERT, the decoder of a transformer has almost as many parameters as BERT. The number of parameters to be learned from scratch is then as large as the number of pretrained parameters, which makes finetuning the model difficult and often too unbalanced to provide good results. Nevertheless, it is possible to reuse BERT for machine translation in a more suitable way, by training in two steps. This is what is proposed in RecycleBERT ^20^ ( Figure 3):

1. Training the decoder alone, with all BERT parameters frozen. This allows training the decoder parameters that have never been trained before and for which a simple finetuning with a low learning rate would not be enough. At the same time, computing resources are saved;
2. Finetuning of the whole model, which can now be done in order to optimise all the parameters of the model, including those of BERT.

In order to generate code from a natural language instruction, a model must be able to capture the meaning of this instruction and to provide a machine-readable representation of it. Code is a formal language, this is why it is preferable to generate inherently valid code by generating grammar production rules, forming an AST. The ASDL formalism allows simply describing ASTs (and how to form them) in a way that is common to all languages.

**Figure 4.**
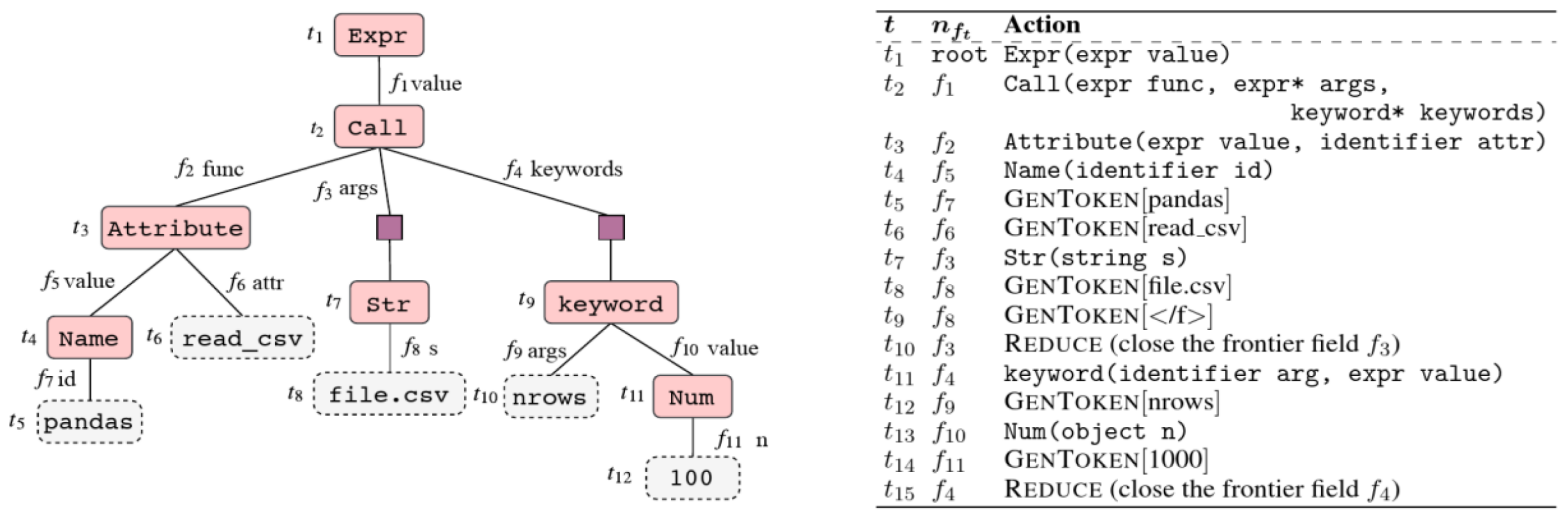
AST of ASDL type of a line of Python code and list of actions generating the tree (From [tranx], under CC-BY).

TRANX is based on a transition system to link an instruction in natural language to an AST, thanks to a series of actions building a part of the tree at each step and using the ASDL grammar ( Figure 4). Once the ASDL AST is fully generated, it is converted into an AST adapted to the target language, and then into source code. These two conversions are easy to perform and can be done with hardcoded functions, simply from the grammar of the target language.

The different actions allowing the generation of the tree are determined in a sequential way by a neural network of type encoder-decoder with attention, the encoder and the decoder being constituted of LSTM cells. The decoder also has a “parent feeding” option, which aims to reflect the topology of the ASTs and thus improves performance when generating code for an object-oriented language. The parent feeding consists in concatenating to the last hidden state a vector *pt,* which encodes the information of the position in the tree.

If models like TRANX already allow obtaining good results for simple code generation problems, it is often difficult to generate correctly a class method when it must call methods or variables already defined within the class. The Concode model should better allow taking into account this “programming context”, through a particular encoder-decoder structure and an innovative training procedure, The corpus used for Concode training is constituted by the authors from more than 33,000 Java projects on GitHub. The datasets are not composed of only *{natural language, code}* pairs. The specificity of this corpus lies in the fact that the training takes into account all member variables and methods of the class from which each example is extracted.

## 4. Results

### 4.1 Variable misuse

This section shows the results obtained for each use case by the implemented model. We use four different metrics for evaluating its performance:

1. **True negative:** Percentage of the bug-free programmes in the ground truth classified as bug free.
2. **Classification accuracy:** Percentage of total programmes in the test set classified correctly as either bug free or buggy.
3. **Localisation accuracy:** Percentage of buggy programmes for which the bug location is correctly predicted by the model.
4. **Localisation + repair accuracy:** Percentage of buggy programmes for which both the location and repair are correctly predicted by the model.

The evaluation of the approach is done over two large test sets composed by 16,334 programmes written in Java, and 2,917 C and C++ source code files.

***4. 1. 1. Java model***

**Figure 5.**
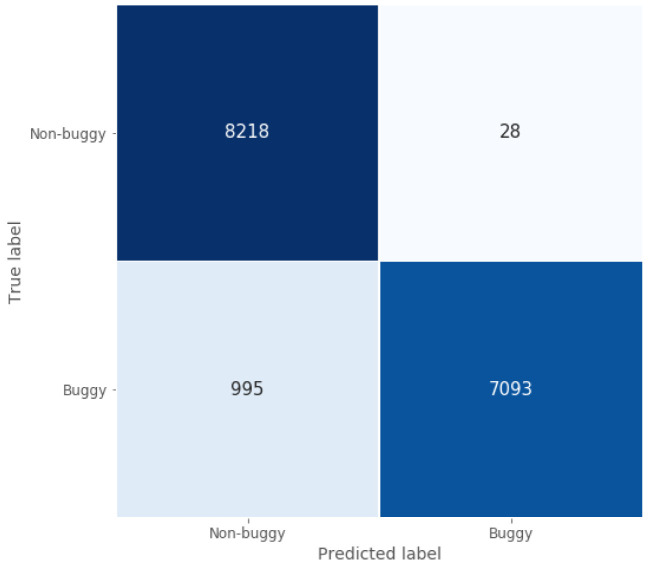
Java model confusion matrix.

When focusing on the classification task, the confusion matrix represented in Figure 5 shows how well the model implemented for this Java use case classifies programmes, since most of the non-buggy and buggy programmes are classified correctly. This conclusion is supported by two of the calculated performance metrics presented in Table 3: true negative rate and classification accuracy, which are around 1 and 0.94 for the test set, respectively.

**Table 3.** Variable misuse results.

Use Cases	Performance metrics			
	True Positive	Classification Accuracy	Localisation Accuracy	Localisation + Repair Accuracy
Java	99.7%	93.7%	88.6%	71.1%
C/C++	99.6%	92.9%	85.4%	60.6%

With respect to the localisation and the repair accuracy, this model is able to point the exact bug location for most of the Java source code files used in this task, achieving a high localisation accuracy around 0.87. Moreover, the the localisation + repair accuracy reaches 0.71, which shows the good repair capability of the model when it comes to fix the located bugs in the faulty programmes analysed.

***4.1.2. C/C++ model***

**Figure 6.**
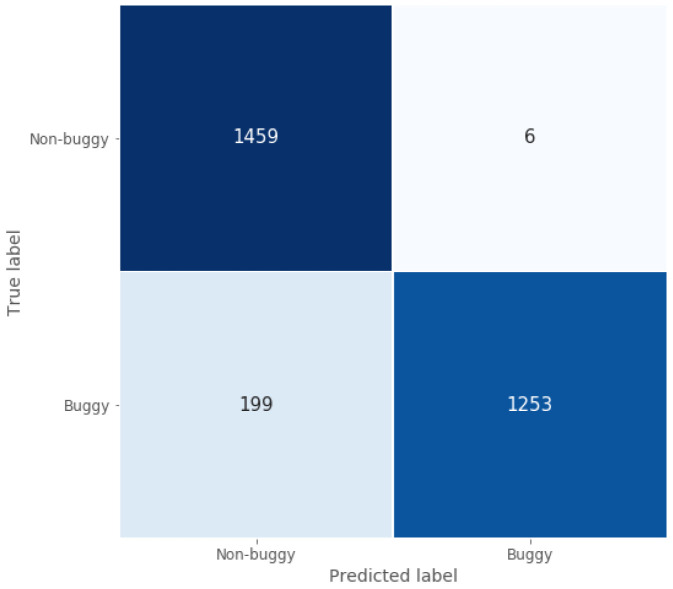
C/C++ model confusion matrix.

Following the same structure as in the previous sub-section, the classification ability of this model can be seen in Figure 6, which shows its good classification performance. Moreover, true negative rate and classification accuracy are high and similar to those obtained by the Java model, since they are around 1 and 0.93 for this C/C++ test set, as shown in Table 3.

The real difference between the two models evaluated in this section lies in their predictions for both the localisation and the repair of the buggy variable. When comparing with the Java use case, the model for C/C++ also reaches a very good localisation accuracy, showing that it is able to predict correctly the localisation of the bug for 85% of the buggy programmes in the test set. However, after locating the error, it repairs a lower percentage of wrong source code files than the Java model, fixing 60% of them, which means approximately 11% less. This may be due to the smaller size of the dataset, being a limitation when training the model.

### 4.2 Code summarisation

This section reports the evaluation of the Java and C/C++ transformers models that have been trained both on DECODER datasets and the augmented datasets versions presented in Section II.

The generated natural language descriptions have been evaluated with two different families of metrics: the BLEU ^37^ score – which is mainly used in machine translation settings – and the ROUGE sets of metrics ^38^ – which are often used to evaluate performances in summarisation tasks:

- Sentence level BLEU_4 score (+ smoothing function)
- Corpus level BLEU_4 score
- SacreBLEU
- ROUGE – L

The BLEU score compares sentences by matching n-grams between the original references and the decoded sentences. Among the BLEU metrics the most adopted version is the BLEU_4, which counts up to 4 n-grams overlap between the generated sentences and the ground truth, although it tends to penalise short sequences assigning a zero value whenever any order n-gram is not encountered. This behaviour has been studied and mitigated with a variety of solutions, some of them involving the adoption of a smoothing function. In this work, the smoothing function (NLTK ‘method4’) is adopted to score the models’ decoded sentences.

On the other hand, the corpus-level BLEU score accounts for the micro-average precision for each hypothesis-reference pair. Being already pondered, no smoothing function is needed when computing this metric. Finally, the sacreBLEU is proposed in 39 and is introduced to overcome the problem of different pre-processing schemes impacting on scores and comparability across models' implementations, by utilising an internal pre-processing.

Besides BLEU scores, belonging to the ROUGE sets of metrics, the ROUGE-L score is also used to assess the quality of the generated descriptions. The L stands for Longest Common Subsequence since the metric computes f1-score, precision and recall by taking into account sentence level structure similarity and identifying the longest co-occurring sequences n-grams.

Below, Table 4 and Table 5 show the results obtained for all the indicated metrics across training, validation and test sets for the models developed upon the use case datasets as well as those obtained with data augmentation. For the Java augmented model, the choice of working with a sample of the original dataset ^9^ has been taken, keeping approximately 300k observations, motivated by the fact that the model covers a single language. Sets were obtained by splitting the datasets according to proportions of (80%-10%-10%) for training, validating and testing, respectively.

**Table 4.** Code summarisation results for the Java models.

	Java DECODER UseCases Model			Java Augmented Model		
Metric	Training	Validation	Test	Training	Validation	Test
**BLEU_4**	83.36	31.70	37.06	51.04	27.82	27.48
**BLEU_4 smooth**	88.61	42.78	47.01	55.50	31.99	31.85
**Corpus BLEU**	93.38	43.85	48.35	58.90	36.04	35.73
**Sacre BLEU**	93.89	44.07	48.53	59.30	36.39	36.18
**Rouge-L Precision**	95.53	58.05	60.80	72.10	51.27	51.20
**Rouge-L Recall**	96.1	60.58	62.77	76.68	55.24	55.31
**Rouge-L F1-score**	95.40	58.61	61.23	73.15	51.64	51.59

**Table 5.** Code summarisation results for the C/C++ models.

	C/C++ DECODER UseCases Model			C/C++ Augmented Model		
Metric	Training	Validation	Test	Training	Validation	Test
BLEU_4	95.98	30.21	26.06	59.27	31.67	32.00
BLEU_4 smooth	97.1	41.51	38.64	66.47	41.54	41.75
Corpus BLEU	97.9	39.45	36.85	71.14	42.20	42.96
sacreBLEU	97.94	39.50	36.87	71.82	42.62	43.03
Rouge-L Precision	98.60	59.51	57.89	80.12	55.65	55.88
Rouge-L Recall	98.79	60.71	58.88	83.51	58.90	59.21
Rouge-L F1-score	98.49	59.63	58.43	80.92	55.87	56.12

***4.2.1. Models’ performances***

Focusing on models built on DECODER datasets, the Java use case model outperforms the version of the C/C++. In contrast to what happens with the Java DECODER model, the performances of the C/C++ drop a little bit by switching from the validation set to the unknown data in the test set.

On the other hand, the augmented models present slightly better performances for C and C++ programming languages, especially for the BLEU set of metrics. With both models, differences between validation and test set performances appear mitigated by switching to the augmented data: in fact, the models score worse on the training set, suggesting that the networks have less tendency to overfitting the training datasets, thus being more robust when compared to the models trained only on DECODER use cases data.

Additionally, the BLEU and ROUGE metrics rely strictly on n-grams appearances, not taking into account sentence meaning or positive model behaviour such as the ability of paraphrasing, cut or enlarge the original descriptions without affecting its meaning and respecting English grammatical rules. Due to this fact, after qualitative analysis and inference experiments were carried out, we reached the conclusion that the augmented models provide a better solution for code summarisation.

That is why these versions are finally going to be integrated into DECODER PKM, due to their greater ability of generalising to new source code methods, thus providing better results in inference settings, despite sometimes producing lower scores in terms of ROUGE or BLEU.

To illustrate this issue, some examples of the generated description from the augmented models are reported below, together with their input methods and original descriptions, with the aim of showing that positive and negative behaviours cannot always be reflected in the evaluation metrics adopted for this task.

***4.2.2. Java examples***

**Figure 7.**
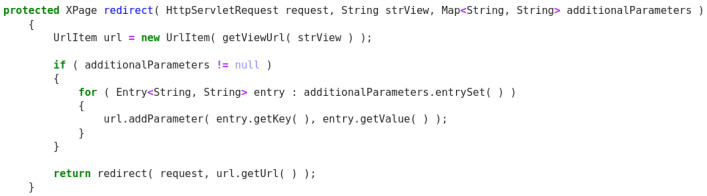
Java snippet - XPage redirect.

- Original description: redirect to an url defined by given parameters
- Predicted description: redirect to another page view
- BLEU_4 smooth: 6.65
- ROUGE-L f1 score: 30.77

In this Java example ( Figure 7), the model produces an acceptable description and can understand that the ‘url’ token that appears in both code and the original descriptions corresponds to a web page. Despite this, having the descriptions only two common tokens, the score cannot reflect a positive behaviour.

**Figure 8.**
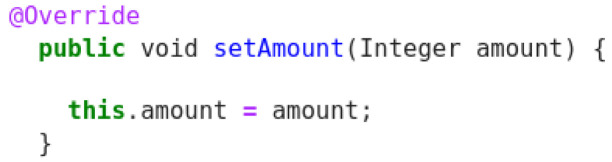
Java snippet - SetAmount.

- Original description: setter for the property type
- Predicted description: getter for the property amount
- BLEU_4 smooth: 29.95
- ROUGE-L f1 score: 60.00

This example ( Figure 8) shows bad quality in the method description, since there is no reference in code to property type. Here the prediction provided by the model captures the reference to amount, although it confuses between setter and getter. Given this, the scores do not really reflect this kind of problem and when compared to other decoded sentences, it receives much higher score than it should get.

**Figure 9.**
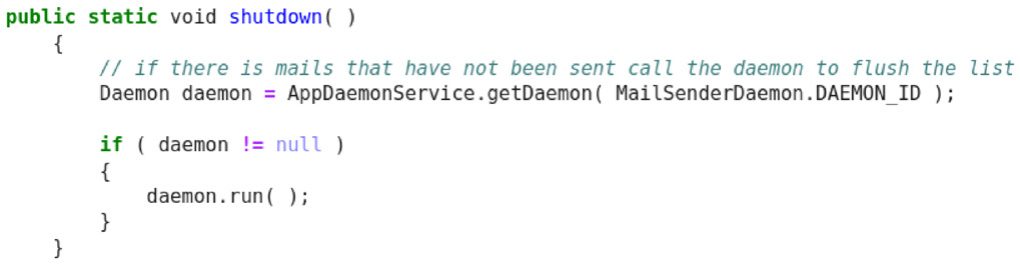
Java snippet - shutdown.

- Original description: shuts down the daemon
- Predicted description: shutdown the service
- BLEU_4 smooth: 6.10
- ROUGE-L f1 score: 28.57

Finally, this example ( Figure 9) shows a very good model behaviour, attending on important tokens in the source code like ‘daemon’ as well as providing a grammatical improvement over the original description by outputting ‘shuts down’. Although the functionality is reflected in the prediction, the tokens do not correspond between the two sentences and a low score is obtained.

***4.2.3. C/C++ examples***

**Figure 10.**
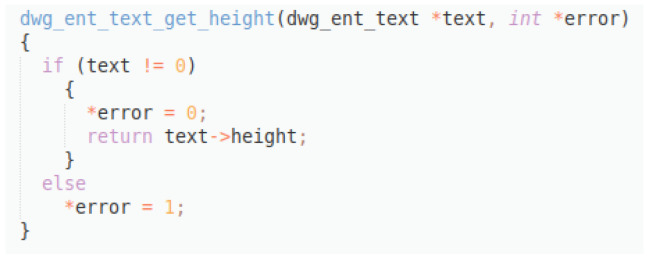
C++ snippet - dwg_ent_text_get_height.

- Original description: usage double height dwg ent text get height text
- Predicted description: returns height of text
- BLEU_4 smooth: 2.08,
- ROUGE-L f1 score: 36.36

In this example ( Figure 10), a good prediction is provided, reflecting the main functionality of the code snippet, although it gets really low scores, especially the BLEU_4, since the original description is not so well formulated. This example reflects the capability of the model to produce acceptable descriptions despite bad data quality.

**Figure 11.**
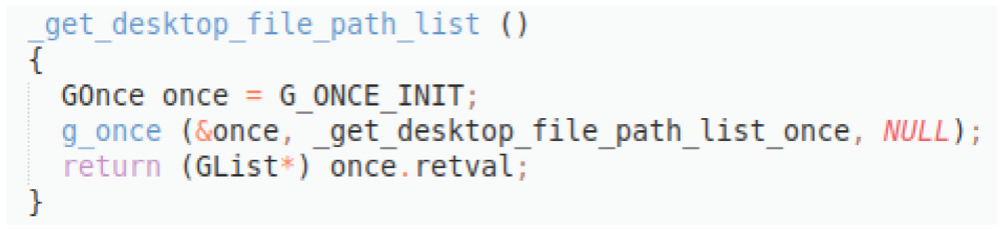
C++ snippet - get_desktop_file_path_list.

- Original description: returns the list of paths to find desktop files
- Predicted description: get desktop file path list
- BLEU_4 smooth: 2.44
- ROUGE-L f1 score: 14.28

Here ( Figure 11), the original description is much better formulated, and the model is able to produce a sentence that reflects the true functionality of the method. However, since the words do not appear with the same frequencies and positions, the scores remain low.

**Figure 12.**
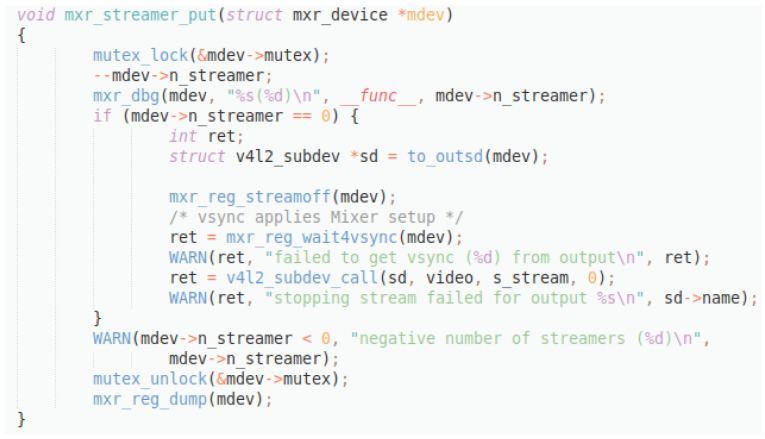
C++ snippet - mxr_streamer_put.

- Original description: removes new client for streaming
- Predicted description: add new client for streaming
- BLEU_4 smooth: 66.87
- ROUGE-L f1 score: 80.00

This last example ( Figure 12) shows an inverse situation: the difference between the original and the predicted description consist in the contradiction of the verb ‘add’ and ‘remove’, while all the other tokens coincide. This situation produces a much higher score, even though the meaning of the descriptions is actually the opposite. 

### 4.3. Semantic parsing

The models of the first category have all been trained on Concode data adapted to context processing by a *seq2seq* model, except for the “RecycleBERT without context” model, which has been trained on the raw pairs *{natural language, code}*. The analysis of the results obtained by each model is performed below:

- Concode: the result provided in Table 6 is the one obtained by the authors of the article. The instructions given did not allow to reproduce it;

**Table 6.** Semantic parsing results.

Language	Model	BLEU 4
Java (Concode)	Baseline: Concode (authors reported result)	22
	Transformer	4
	TRANX	10.1
	RecycleBERT	35
	RecycleBERT without context	30.65
	RecycleCharacterBERT	18.12
C++	RecycleBERT pretrained (OpenCV dataset)	1.5
	RecycleBERT (OpenCV dataset)	4.16
	RecycleBERT (C&C dataset)	14.85

**Figure 13.**
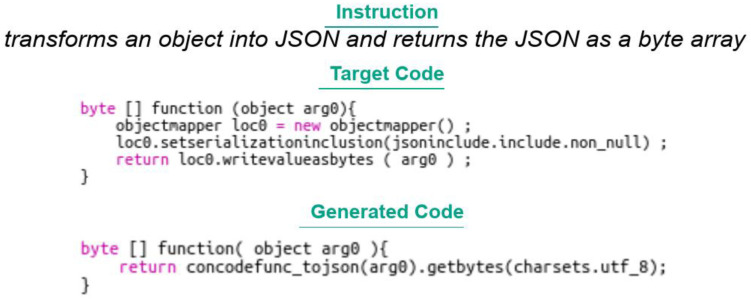
Code from a Concode example associated with an instruction.

- Transformer: The BLEU 4 score obtained by the transformer model developed with PyTorch and its submodules is very low, emphasising the difficulty a classical transformer model has on a task like code generation. It should be noted that beam search has not been used here, which can also explain the poor performance;
- TRANX: The BLEU 4 score is rather weak, which shows the limits of the model in term of performance, in spite of the complete treatment of the JAVA grammar;
- RecycleBERT: The performance obtained by RecycleBERT is much better than the results obtained by Concode authors. This can be explained on the one hand by the use of a recent powerful model and the usage of the information contained in the pretrained embeddings of BERT. However, we notice that the code produced in output is often not syntactically correct, which suggests that taking into account the syntax of the language within such a model would allow to improve its performance. Finally, there is a marked difference between the score obtained by taking into account the context (concatenated with the natural language instruction) and the score without context, which shows that the model is able to capture and reuse certain information from the context (such as method names, for example) while distinguishing them correctly from the rest of the natural language instruction. If the model were unable to make this difference, performance would be impaired. An example is provided in Figure 13. We can see that the model did not succeed in providing the expected result in this particular case, but that the instruction was understood;

- RecycleCharacterBERT: By using a pretrained CharacterBERT model instead of a pretrained BERT, RecycleBERT (logically renamed RecycleCharacterBERT) gives a significantly lower BLUE, which does not follow our intuition. Indeed, the use of CharacterBERT (reasoning at characters forming each word level to determine its embedding) should make it possible to provide more adapted embeddings when working on a very specific corpus, including many terms (and subterms) that are rare and therefore absent from the BERT vocabulary. We have put forward several hypotheses, to be verified in the future, to explain the difference between the expected performance and the actual performance. The first one explains the difference by a potential overestimation of the number of rare words in the natural language instructions, which would lead to a better performance of BERT, its embeddings being more accurate than those of CharacterBERT on a text containing few words outside the vocabulary. The second hypothesis would explain the decrease in performance simply by an incorrect adaptation of the structure of RecycleBERT for CharacterBERT. This result should be considered as temporary.

The training of RecycleBERT on C++ corpora has globally given poorer results than on Concode, which is probably explained by the difference in data quality. On the first corpus (OpenCV), the model was trained in two different ways. Indeed, the corpus being quite small, we first tried to simply finetune on the OpenCV corpus the model trained on Concode, hoping that the similarities between JAVA and C++ would be enough for the model to learn to generate C++ on a small amount of data. The obtained BLUE score (1.5) being very low, to the point of suggesting that the model has in fact not learned anything, we tried to train RecycleBERT only on the OpenCV corpus. The results obtained are a little bit better (4.16), showing the failure of knowledge transfer between JAVA and C++, but are still very low. The small size of the corpus is therefore not compatible with training a model like RecycleBERT. However, training on the C&C corpus gives a BLEU of 14.85. The difference in performance compared to training on the OpenCV corpus is undoubtedly due to the much larger size of the C&C corpus. The important difference between the BLEU obtained on C&C and the one obtained on Concode is explained by the difference in quality between the two datasets, the code comments being necessarily less precise and noisier than the code generation instructions. This lower data quality also leads to more difficult learning of syntax rules. We have not evaluated the part of the generated code that could be syntactically correct but it is clear that it is quite low with the current model. This also suggests that performance could be greatly improved by adapting the model to generate syntactically correct code. But the C++ syntax is a lot more complex than the Python or even Java ones.

## 5. Conclusions

As more and more aspects of life depend on the reliable operation of high-quality software, the objective of this work is to present some new tools that help to improve IT professionals’ productivity by facilitating their daily work. In particular, this paper presents and describes three distinct tools based on NLP techniques: variable misuse, code summarisation and semantic parsing.

The variable misuse problem is a type of error which refers to the wrong location of variables within source code that affects the correct behaviour of the programme, causing failures in it. Several works have focused on this common problem, most of them present learning-based repair solutions that learn how to fix this class of error directly from source code examples. The method we have presented in this paper makes use of one of them based on a novel technique called pointer networks and extends it to other programming languages historically more used. Therefore, with the objective of alleviating debugging tasks of software developers, we take advantage from using deep learning end-to-end models to locate and repair faulty variables contained in source code files written in Java, C or C++. After an evaluation over two different datasets, we have shown how our tool is able to correctly classify about a 93% of Java, C and C++ files, which shows the effectiveness of the approach for this classification task. However, there are some differences in the bug-repair ability of both models. The Java model outperforms the model implemented for C/C++ use case, since it is able to correctly locate and repair the bug for roughly 71% of the buggy Java programmes, whereas the C/C++ model only manages to repair a 60% of these. This difference is probably due to the smaller amount of training data of the C/C++ use case, even when this dataset size was increased with the incorporation of new source code files from third-party projects.

Focusing on the code summarisation tool, the results obtained are quite good for all the programming languages involved in our experiments. In this conclusion, when we refer to our best models, we are always referring to the augmented versions of the models developed for Java and C/C++. Although Java has been the object of various studies within code summarisation, it is difficult to define a baseline reference due to the different metrics (and even versions of them) that are used in different studies that make the assessment uncertain. The resulting metrics for our Java model highlight that we went beyond the state of the art when focusing on metrics such as the ROUGE-L, whereas the performances are lower than the state of the art when evaluating with multiple versions of the BLEU score, emphasising the ambiguity that exists when it comes to evaluate models developed for this task. On the contrary, to our knowledge, the C and C++ languages have not been explored for code summarisation yet. This means that our code summarisation model for C/C++ proposes a new application for this task based on new languages, consequently having no terms of comparison for the performances of our models. Given this, we observed that our model for C/C++ outperforms the Java models in both sets of metrics, suggesting that it consists in a valid solution to automatically generate descriptions for C and C++ source code methods.

However, there is room for improvement of the code summarisation tool, taking into consideration that it is a quite new field of investigation and that no experiments have been conducted for C and C++ languages so far. Solutions like trying different pre-processing techniques and code representations (like AST based representations) or architectures (such as graph neural networks) could help in increasing the performances. Moreover, the usage of a pre-trained model to calculate domain specific embeddings could be useful to achieve better results for this task.

Considering semantic parsing, the main goal of this work was to go beyond the results of the state of the art in code generation, by improving existing models with the latest advances in NLP. The development of a model aggregating these various improvements has been undertaken. Its architecture seems now clearly defined. However, its implementation is still in progress. Nevertheless, during the research process linked to the development of this model, several machine translation and code generation models were evaluated on the available data sets. This allowed highlighting the efficiency of the most recent NLP models, able to obtain higher scores than the state of the art in code generation of 2018, without any mechanism ensuring the production of syntactically correct code. The inclusion of such a mechanism in the model currently under development allows us to expect improved performance. We can highlight three main components in a competitive model: the use of the most recent machine translation models using pretrained embeddings such as those of BERT, a mechanism such as TRANX based on ASTs and ASDL allowing to generate syntactically correct code, and taking into account of the context of the class within which the code must be generated. This last aspect is not optimally handled by our current model, which simply concatenates the context to the natural language instruction. This leaves room for possible improvements of the model in the future.

## Data availability

### Underlying data

The datasets corresponding to the use cases considered in this paper are collected in public repositories:

Zenodo: DECODER OpenCV use case data. https://doi.org/10.5281/zenodo.4333362 ^26^.

- OpenCV datasets for DECODER project deliverable D6.2 "Use-case data from the PKM". See the deliverable for further details (deliverable is available on project website).

Zenodo: OW2 Decoder java use-cases data (WP6). https://doi.org/10.5281/zenodo.4322471 ^27^.

- OW2 data for use-cases Authzforce, Joram, Lutece, Sat4j.

Zenodo: Training dataset for the Drivers use case. https://doi.org/10.5281/zenodo.5780179 ^28^.

- Training dataset for the Drivers use case (C language) obtained from the "excavator" dataset https://zenodo.org/record/4383876 (simplified version of the original linux code).

https://github.com/devonfw/my-thai-star

- MyThaiStar is the reference application that Capgemini uses internally to promote best programmeming practices and the correct use of last technologies. It is developed with Devon Framework, the standard tool for development at the company.

Data are available under the terms of the Creative Commons Attribution 4.0 International (CC-BY 4.0).

In addition, this section includes the databases that contain the different datasets/source code files used for augmenting data. For the variable misuse tool, we extracted some code files from these GitHub repositories:

- https://github.com/ros-perception/vision_opencv (accessed on 27/01/2021)
- https://github.com/Selameab/icog_face_tracker (accessed on 27/01/2021)
- https://github.com/chrissunny94/Lane_Detection (accessed on 27/01/2021)
- https://github.com/tzutalin/ros_sample_image_transport (accessed on 27/01/2021)
- https://github.com/qutas/kinetic_sample_packages (accessed on 27/01/2021)
- https://github.com/ros/ros_tutorials (accessed on 27/01/2021)
- https://github.com/introlab/find-object (accessed on 27/01/2021)
- https://github.com/joselusl/aruco_eye (accessed on 27/01/2021)
- https://github.com/ros-perception/image_pipeline (accessed on 27/01/2021)
- https://github.com/laurentkneip/opengv/tree/master/src/relative_pose (accessed on 27/01/2021)
- https://github.com/Longpham3105/learnopencv.com (accessed on 27/01/2021)
- https://github.com/PacktPublishing/-OpenCV-By-Example (accessed on 27/01/2021)
- https://github.com/oreillymedia/Learning-OpenCV-3_examples (accessed on 27/01/2021)
- https://github.com/nrsyed/computer-vision/tree/master/ColorThreshUtil (accessed on 27/01/2021)
- https://github.com/tobybreckon/cpp-examples-ipcv (accessed on 27/01/2021)

Regarding the code summarisation task, the Code and Comments dataset was used to extract observations to augment data, which can be found in this repository:

- Zenodo: Code and Comments Dataset. https://doi.org/10.5281/zenodo.3472050 ^31^.

The code and comment data are a compilation of code blocks and their related comments. Doxygen successfully ran on 106,304 different GitHub projects. A total of 16,115,540 code- comment pairs were obtained by running Doxygen on C, C++, Java, and Python projects.

- https://github.com/xing-hu/DeepCom (accessed on 17/05/2021)

Finally, we include the repository that contains the “Concode Dataset” used in the experiments of the semantic parsing tool, although the data used in this case was also augmented using the “Code and Comments Dataset” mentioned above:

- https://github.com/sriniiyer/concode (accessed on 18/08/2020)

Data are available under the terms of the Creative Commons Zero “No rights reserved” data waiver (CC0 1.0 Public domain dedication).

## Software availability

**Source code available from:**

- https://gitlab.ow2.org/decoder

**Archived source code at time of publication:**

- Zenodo: Variable misuse tool (ORE). https://doi.org/10.5281/zenodo.6034599 ^23^.
- Zenodo: Code Summarization tool (DECODER project). https://doi.org/10.5281/zenodo.6090276 ^24^.
- Zenodo: Semantic parser tool from the Decoder project. https://doi.org/10.5281/zenodo.6280897 ^25^.

**License:**

- GNU Affero General Public License v3 (AGPL-3.0)

## Ethics and consent

Ethical approval and consent were not required.
