# Supplementary material for: An open-source natural language processing toolkit to support software development: addressing automatic bug detection, code summarisation and code search
Source: Open Res Eur. 2023 Oct 27;2:37. Originally published 2022 Mar 14. [Version 2] doi: 10.12688/openreseurope.14507.2 (PMC11036033; doi:10.12688/openreseurope.14507.2)
Supplement: Peer review history

# Reviewer report: Reviewer response for version 2

**DOI:** 10.21956/openreseurope.18099.r39237

**Published:** 2024-04-22

**Authors:** Nan Jiang

**Affiliations:** 1 Computer Science Department, Purdue University, West Lafayette, Indiana, USA

This work aims to integrate NLP techniques into the Persistent Knowledge Monitor (PKM) framework. The PKM acts as a comprehensive knowledge base, supporting queries and analyses through its meta-model. It is accessible via a user-friendly interface tailored to different user roles—developers, maintainers, and reviewers—each able to interact with the PKM using personal accounts. This setup facilitates an organized and efficient approach to handling complex software development tasks.

 Specifically, during the development, this work develops and evaluates several NLP techniques for three crucial tasks, focusing on variable misuse, code summarization, and semantic parsing.

 For variable misuse, this work presents a learning-based encoder-decoder repair solution employing pointer networks to identify and correct variable misuse in Java, C, and C++ code. This model has shown a high classification accuracy of approximately 93% across these languages. For bug-repair ability, the presented approach shows higher accuracy on Java language than C/C++, which likely due to the lack of enough C/C++ training data.

 For the code summarization task, this work presents the transformer-based approach, which demonstrates promising results on all programming languages, especially with Java and C/C++. Besides, the paper suggests surpassing current benchmarks with metrics like ROUGE-L. And for C and C++, where prior research is lacking, the tool sets a new standard, showing superior performance in generating method descriptions, indicating a robust model despite the lack of comparative benchmarks.

 For semantic parsing, this work presents several techniques such as Concode, TRANX, RecycleBERT, and a modified RecycleBERT (replacing the pretrained BERT with a pretrained CharacterBERT). While the full implementation of the new models is still underway, preliminary tests with existing machine translation and code generation models show promising results that exceed the state of the art from 2018. The future model aims to incorporate features like syntax correctness and better context integration.

Is the rationale for developing the new method (or application) clearly explained?

Yes

Is the description of the method technically sound?

Yes

Are the conclusions about the method and its performance adequately supported by the findings presented in the article?

Yes

If any results are presented, are all the source data underlying the results available to ensure full reproducibility?

Yes

Are sufficient details provided to allow replication of the method development and its use by others?

Yes

Reviewer Expertise:

Software Engineering, Natural Language Processing, Large Language Model

I confirm that I have read this submission and believe that I have an appropriate level of expertise to confirm that it is of an acceptable scientific standard.

# Reviewer report: Reviewer response for version 2

**DOI:** 10.21956/openreseurope.18099.r35896

**Published:** 2023-11-03

**Authors:** Miltos Allamanis

**Affiliations:** 1 Google DeepMind, London, UK

The comments from my previous review have been adequately addressed.

Is the rationale for developing the new method (or application) clearly explained?

Partly

Is the description of the method technically sound?

Yes

Are the conclusions about the method and its performance adequately supported by the findings presented in the article?

Yes

If any results are presented, are all the source data underlying the results available to ensure full reproducibility?

Yes

Are sufficient details provided to allow replication of the method development and its use by others?

Yes

Reviewer Expertise:

machine learning

I confirm that I have read this submission and believe that I have an appropriate level of expertise to confirm that it is of an acceptable scientific standard.

# Reviewer report: Reviewer response for version 1

**DOI:** 10.21956/openreseurope.15660.r29626

**Published:** 2022-09-12

**Authors:** Abdelwahab Hamou-Lhadj

**Affiliations:** 1 Concordia University, Montreal, Canada

The authors present Persistent Knowledge Monitor (PKM), a framework that uses NLP to aid in software development and maintenance tasks. The framework targets three tasks: automatic detection and localization of variable misuse, code summarization, and semantic parsing.

 The rationale for developing PKM is clearly stated. Using AI in software maintenance  can help reduce the amount of time and effort required to maintain large software systems. This time can be used to create interesting features that provide value to customers.

 The selection of the three tasks that developers can perform with the framework appears odd. While variable misuse is a significant issue, it is only a minor aspect of overall code quality. Code summarization is needed for programme comprehension, which in turn helps in the detection and correction of bugs. Semantic parsing is not directly related to software maintenance and is not something that many developers would require in order to understand and debug code, the framework's main purpose.

 Furthermore, the following points in the article require clarification:

- The description of the datasets used for the three tasks is a little hazy. For the variable misuse case study, for example, the authors simply state the projects' name and the number of files they contain.  It would be better if the authors included more details, such as the number of lines of code, the number of years the systems have been in operation, and so on, to help the reader have an idea of the complexity of these systems. I have a similar comment about the dataset used for code summarization. The authors stated that an existing C/C++ dataset was augmented (Section 3.1.3), but they do not explain how this was done. The number of systems included in this dataset was not provided either.
- Many of the presented algorithms in the model implementation section require the setting of hyperparameters, which is not covered in the article. The authors mention various DL algorithms and NLP pre-trained models, but do not go into detail about how these algorithms are used to run the experiments presented in the paper, particularly when finetuning the models.
- For the variable misuse case study, the number of synthetic buggy files generated is significantly greater than the number of healthy files (e.g., for Java, there are 8,058 healthy files compred to 54,448 buggy files). Wouldn't this result in a data imbalance issue? How did the authors handle this?
- In the Results section on variable misuse, the authors presented four evaluation metrics, which, in my opinion, are insufficient to understand the tool's performance. For example, classification accuracy, which is the percentage of total programmes in the test set correctly classified as bug free or buggy, is not useful. It would have been better to report the percentage of bug-free programmes that are correctly classified as well as the number of buggy programmes that are correctly classified as two separate metrics. Indeed, I believe the authors should employ traditional evaluation metrics such as true positives, false positives, true negatives, and false negatives, as well as accuracy, precision, recall, and f1-score. I'd also recommend including the AUC. Furthermore, I do not believe we should combine the results of variable misuse detection and variable misuse localization. These are two distinct classification problems, and the results should be reported separately (using the aforementioned metrics).
- The code summarization examples are interesting. Perhaps, the authors should provide examples for the two other tasks (variable misuse and semantic parsing) as well.

Other thoughts:

- I recommend including a process that explains how the proposed framework should be used in practice by software developers and maintainers, especially given the number of algorithms involved.
- Because these tools use DL algorithms, I recommend discussing the ethical dimensions (if any) as well as the AI algorithms' explainability. AI research should always include these aspects, in my opinion, to increase confidence in using these techniques.

Is the rationale for developing the new method (or application) clearly explained?

Yes

Is the description of the method technically sound?

Partly

Are the conclusions about the method and its performance adequately supported by the findings presented in the article?

Yes

If any results are presented, are all the source data underlying the results available to ensure full reproducibility?

Partly

Are sufficient details provided to allow replication of the method development and its use by others?

Partly

Reviewer Expertise:

Software Engineering, Software Maintenance and Evolution, Software Tracing and Logging, AIOps, Anomaly Detection, Model-Driven Engineering.

I confirm that I have read this submission and believe that I have an appropriate level of expertise to confirm that it is of an acceptable scientific standard, however I have significant reservations, as outlined above.

# Response

**Published:** 2023-10-17

**Authors:** Cristian Robledo

**Affiliations:** Treelogic, Spain

First of all, thanks a lot for your time and your review. I will try to respond you following the same structure you used for your comments.

- We have included more information about the datasets to the 2nd version of the work, which has already been submitted. We have added information regarding the total number of lines for each dataset. However, we do not really know what you mean with “systems”. If you refer to the numer of years that these applications have been working or in maintenance, we think this information is not strictly necessary for the reader and the understanding of the tools/models we present in our work. Concerning the data augmentation on the C/C++ use case in code summarization task, we just joined the DECODER dataset with the augmented dataset (also known as “Code and Comments Dataset”) together and ran the corresponding model. We hope this answer your question.
- The hyperparameters finally selected and used for every model have been included in Results section in the 2nd version of the article. Regarding the Deep Learning models, we think that the information we provide to the readers is enough to understand how they work. One of the reasons is that they can turn to the original works of the models if they want to go into detail. These works are properly indicated in our article in order to facilitate their access.
- The dataset for the VarMisuse task is not imbalanced. Following your example, you are right: we use those 8k healthy Java files to automatically create 54 buggy files. But in this process we also include an original copy for each buggy file created, so at the end of the stage we have 54k (healthy) original files and 54 buggy files, which ensures a 50/50 balance. This information is already provided in Subsection 3.2.1.
- It is true that more traditional metrics such those that you comment could be use for the evaluation of models, buy they are not really related to the purpose of VarMisuse task, which is to locate and repair wrong uses of variables in programmes. However, we provide a classification matrix of the test set for each use case (Java and C/C++) , so most of the metrics mentioned in your comment can be directly calculated for anyone who wants that information. Moreover, these traditional metrics are more related to the VarMisuse detection (in case we state this problem as the detection of a wrong use of a variable in a source code file) rather than VarMisuse localisation and repair. Then, VarMisuse detection can be seen as a means for achieving the VarMisuse localisation, because it helps to refine our purposed task by pointing out those files which will need the fixing of the code later. So both classifications work better together, at least for our objective.
- We have included a couple of Figures to illustrate the VarMisuse task in the Result section in the 2nd version of the article, since an example of Semantic Parsing task was already provided.

# Reviewer report: Reviewer response for version 1

**DOI:** 10.21956/openreseurope.15660.r28824

**Published:** 2022-03-16

**Authors:** Miltos Allamanis

**Affiliations:** 1 Google DeepMind, London, UK

This work replicates deep learning research on three software development tasks (variable misuse, code summarisation, and semantic parsing) for Java, C, and C++.

 This work seems technically correct and adds value to the research community by showing how a set of methods can be consistently applied to support software development, especially in C, C++ which have been relatively neglected in the community. However, its methodological novelty is unclear since no new methods are developed but replications of existing machine learning models are used for C/C++ and Java. Specifically:

- VarMisuse follows the method of Vasic *et al.* (2019) [8]
- Code summarisation follows Ahmad *et al.* (2020) [5]
- Semantic Parsing is a variation of Imamura *et al.* (2019) [20]

 In that sense, I believe this work should probably *not *be listed as a "method article". An alternative classification is probably more appropriate here.

**High-level comments**

- The title eludes to a "toolkit" but the paper looks like a disparate set of three tools/machine learning models. What are the (common) aspects that make this a "toolkit"? How are these tools applicable in a broad set of scenarios?
- The choice to use a relatively small dataset (DECODER) where possible, is interesting and different from most work that focus on large datasets. This should be better highlighted and contrasted.

**Related Work**

 Some related work is missing and would be useful to be discussed and contrasted. Some comparison with the evaluation section might also be useful:

- VarMisuse
  - Pradel, Michael, and Koushik Sen. "Deepbugs: A learning approach to name-based bug detection." Proceedings of the ACM on Programming Languages. OOPSLA (2018). ^1^
  - Hellendoorn, Vincent J., *et al*. "Global relational models of source code." International conference on learning representations. 2019. ^2^
  - Allamanis, Miltiadis, Henry Jackson-Flux, and Marc Brockschmidt. "Self-Supervised Bug Detection and Repair." Advances in Neural Information Processing Systems (2021). ^3^
- Code Summarisation
  - Parvez, Md Rizwan, *et al.* "Retrieval Augmented Code Generation and Summarization." Findings of the Association for Computational Linguistics: EMNLP 2021. 2021. ^4^
  - Clement, Colin, *et al.* "PyMT5: multi-mode translation of natural language and Python code with transformers." Proceedings of the 2020 Conference on Empirical Methods in Natural Language Processing (EMNLP). 2020. ^5^
  - Haque, Sakib, *et al.* "Improved automatic summarization of subroutines via attention to file context." Proceedings of the 17th International Conference on Mining Software Repositories. 2020. ^6^
  - Lu, Shuai, *et al.* "CodeXGLUE: A Machine Learning Benchmark Dataset for Code Understanding and Generation." Thirty-fifth Conference on Neural Information Processing Systems Datasets and Benchmarks Track (Round 1). 2021. ^7^
- Semantic Parsing
  - Chen, Mark, et al. "Evaluating large language models trained on code." arXiv preprint arXiv:2107.03374 (2021). ^8^
  - Lu, Shuai, et al. "CodeXGLUE: A Machine Learning Benchmark Dataset for Code Understanding and Generation." Thirty-fifth Conference on Neural Information Processing Systems Datasets and Benchmarks Track (Round 1). 2021.

 
**Technical Comments**

- Variable Misuse:
  - "after the search on GitHub the number of source code files…". It's unclear what was searched on GitHub? Why "just" ~700 files were found/added to the dataset?
  - The dataset split is unclear: does each file (and its buggy versions) appear in the same train/validation/test fold? Please, clarify.
  - The generated bugs may be "too easy" to detect or can be detected by the relevant compiler (Sec 3.1). For example, variable misuses that lead to type checker errors or use-before-def errors. Have the authors filtered these out?
  - The performance on the VarMisuse task seems much better than the one reported by Vasic et al. What explains this difference? One potential theory is that the generated bugs are "too easy" (see bullet above). Discussing any potential explanations for this in the paper would be very useful.
- Code Summarisation
  - Some additional information on the inputs to the transformer model would be useful. Specifically, the vocabulary creation (is BPE used, for example?), tokenization, etc.
- Semantic Parsing
  - It's unclear if the "classical transformer model"  includes positional encodings (the PyTorch nn.Transformer doesn't incorporate them automatically).
  - Although using BLEU4 is a plausible metric, alternatives have been shown to correlated better with the task, e.g. Ren, Shuo, et al. "Codebleu: a method for automatic evaluation of code synthesis." arXiv preprint arXiv:2009.10297 (2020). ^9^ Please consider reporting them too. 

**Minor Comments**

- A citation to Vaswani *et al.* (2017) ^10^ for Figure 2 would probably be appropriate.
- Fig 5 and Fig 6 could be smaller or the text (numbers) larger.

Is the rationale for developing the new method (or application) clearly explained?

Partly

Is the description of the method technically sound?

Yes

Are the conclusions about the method and its performance adequately supported by the findings presented in the article?

Yes

If any results are presented, are all the source data underlying the results available to ensure full reproducibility?

Yes

Are sufficient details provided to allow replication of the method development and its use by others?

Yes

Reviewer Expertise:

machine learning

I confirm that I have read this submission and believe that I have an appropriate level of expertise to confirm that it is of an acceptable scientific standard, however I have significant reservations, as outlined above.

## References

[1] : DeepBugs: a learning approach to name-based bug detection. *Proceedings of the ACM on Programming Languages* .2018;2(OOPSLA) : 10.1145/3276517 1-25 10.1145/3276517

[2] : Global relational models of source code. *International conference on learning representations* .2019; Reference source

[3] : Self-Supervised Bug Detection and Repair. *Advances in Neural Information Processing Systems* .2021; Reference source

[4] : Retrieval Augmented Code Generation and Summarization. *Findings of the Association for Computational Linguistics: EMNLP 2021* .2021; 10.18653/v1/2021.findings-emnlp.232 10.18653/v1/2021.findings-emnlp.232

[5] : PyMT5: multi-mode translation of natural language and Python code with transformers. *Proceedings of the 2020 Conference on Empirical Methods in Natural Language Processing (EMNLP)* .2020; 10.48550/arXiv.2010.03150 10.48550/arXiv.2010.03150

[6] : Improved automatic summarization of subroutines via attention to file context. *Proceedings of the 17th International Conference on Mining Software Repositories* .2020; 10.1145/3379597.3387449 10.1145/3379597.3387449

[7] : CodeXGLUE: A Machine Learning Benchmark Dataset for Code Understanding and Generation. *Thirty-fifth Conference on Neural Information Processing Systems Datasets and Benchmarks Track (Round 1)* .2021; 10.48550/arXiv.2102.04664 10.48550/arXiv.2102.04664

[8] : Evaluating large language models trained on code.2021; 10.48550/arXiv.2107.03374 10.48550/arXiv.2107.03374

[9] : Codebleu: a method for automatic evaluation of code synthesis.2020; 10.48550/arXiv.2009.10297 10.48550/arXiv.2009.10297

[10] : Attention Is All You Need. *Proceedings of the 31st International Conference on Neural Information Processing Systems* .2017; 10.48550/arXiv.1706.03762 10.48550/arXiv.1706.03762

# Response

**Published:** 2023-10-17

**Authors:** Cristian Robledo

**Affiliations:** Treelogic, Spain

First of all, thanks a lot for your time and your review. I will try to response you following the same structure you used for your comments. Regarding the classification, we agree with you about perhaps "Method Article" is not the most appropriate tag for the article. However, given the alternatives available in the "Engineering and technology" subject area, we selected this tag because we thought that it is more suitable than the others at least. We also thought about classifying the paper as "Software Tool Article", but the problem is that we (the authors) did not work on the integration and deployment side of the whole tool (known as Persistent Knowledge Monitor, or PKM), so we lack most of the information to develop and implement the toolkit from scratch, as it should be stated in a "Software Tool Article".  

**High-level comments**

- We use the term "toolkit" because they belong to a set of tools integrated in the PKM that aim to facilitate the daily work of software developers and mantainers. In the article, we try to emphasise the development of these three Natural Lanugage Processing tools rather than the synergies existing between them. However, they exist: Imagine using this set of tools at a single project level. In case a new person joins the project with no idea about how it is its architecture/structure or how its code works, he/she could use some of this tool in order to understand, fix and maintain any programme file of the project. Code summarization could help him by giving short explanations for those undocumented functions/methods, while Varmisuse could alleviate his workload by fixing most minor errors related to wrong variables localisation or use. Perhaps the most disparate tool is the Semantic Parsing one, but it could also be used as an introduction tool to the project, because it could help to code easily in a fast way with no need of having a complete knowledge of the project.
- The response of this comment has been included in the 2nd version of the work, which has already been submitted.

**Related Work** We have already had most of the works you mention in mind in previous stages of the study. However, these have not been included in the article since we did not want it to be too long. We have opted for including just the most related works in our paper. **Technical Comments**

- Variable Misuse:
  - All the replies to these comments have been already included in the 2nd version of our work in their corresponding sections. Please, check them.

- Code Summarisation
  - We have an specific section explaining this that we have extended in this 2nd version of the work. Please, check 3.2.2.

- Semantic Parsing
  - Yes, positionnal encoding was used (https://github.com/kleag/transformer_NMT/blob/944f45ffe911a706cf7de40b2ec0c3e9bb0f1db1/transformer_model/translation_script_concode.py#L149), so the sentence can be rewritten as "classical transformer model with positionnal encoding". However, we miss do this in the 2nd version. Hopefully this comment serves as additional information for readers.
  - Yes, BLEU4 is not able to judge the quality of produced code, just its similarity at tokens level with the reference. Our future work will be evaluated with more recent metrics dedicated to code, like "Codebleu" and test-oriented benchmarks like HumanEval-X, but we think it is not necessary for the purpose of this paper, which is more generalist.
